# Association between exercise-related social media use and exercise self-efficacy: a PLS-SEM approach

**DOI:** 10.3389/fpubh.2026.1895806

**Published:** 2026-07-13

**Authors:** Yueyang Mao, Jiyuan Zhao, Meng Xiang, Haowei Huliang, Wei Zhou

**Affiliations:** 1 College of Physical Education, Sichuan University of Science & Engineering, Zigong, China; 2 School of Journalism and Communication, Chongqing Jiaotong University, Zigong, China

**Keywords:** exercise self-efficacy, exercise-related social media use, peer relationships, psychological capital, psychological resilience, social capital

## Abstract

In the era of digital media, social media has become a crucial platform shaping college students' exercise behaviors. However, the structural pathways linking exercise-related social media use and exercise self-efficacy remain largely unresolved, particularly regarding how external digital interactions translate into internal psychological resources. Integrating social capital and psychological capital within the Social Cognitive Theory framework, this study explores the structural and indirect associations between exercise-related social media use and exercise self-efficacy. Using a cross-sectional survey design, data were collected from a valid sample of 385 Chinese college students. Partial Least Squares Structural Equation Modeling (PLS-SEM) was employed to evaluate a structural model incorporating peer relationships and psychological resilience, while the out-of-sample predictive validity was assessed via the PLSpredict procedure. The findings reveal that exercise-related social media use has no significant direct association with exercise self-efficacy; rather, it exhibits a significant overall positive relationship (β = 0.196). This overall path operates primarily through indirect pathways, with a significant sequential indirect pathway from peer relationships to psychological resilience (β = 0.081, *p* < 0.01). These results indicate that digital contextual resources are structurally linked to self-efficacy through sequential social and psychological factors. This study demonstrates that external digital resources must be embedded within social relationships and internalized as individual psychological capital to be genuinely associated with behavioral beliefs. Consequently, university health interventions should shift their focus from mere information provision to fostering supportive environments that build peer connections and psychological resilience.

## 1 Introduction

In recent years, with the rapid development of digital media technology, social media has gradually become an important platform for college students to access information, interact with others, and shape their lifestyles (1–3). Previous research has shown that sports content on social media can inform individuals' perceptions of exercise and behavioral tendencies through dynamics like social comparison, vicarious experience, and group interaction. However, there remains disagreement regarding the exact nature of these relationships (4). At the individual level, exercise self-efficacy—a key psychological factor predicting physical activity participation—has been widely shown to play a crucial role in initiating and maintaining exercise behavior (5, 6). Based on Albert Bandura's social cognitive theory, there is a dynamic interplay between individual behavior, cognitive factors, and environmental factors, with self-efficacy serving as the core psychological bridge connecting external circumstances to behavioral outcomes (7). Recent evidence suggests that digital and technology-supported physical activity promotion is increasingly moving beyond information delivery toward personalized, adaptive, and behaviorally informed intervention models (8). However, digital exposure may not directly translate into exercise-related confidence because physical activity remains shaped by effort-related barriers and motivational inertia (9).

Because simple exposure cannot automatically overcome these inherent barriers, the structural relationship between exercise-related social media use and exercise self-efficacy becomes highly complex. Consequently, it is necessary to clearly distinguish between its two behavioral sub-dimensions: passive content consumption (e.g., scrolling through fitness influencers' videos or watching peers' daily workout routines) and active social interaction (e.g., posting workout updates, liking, and commenting). On the one hand, active social interaction transforms users from mere recipients of information into active community participants. The atmosphere of sports participation fostered by community activities—such as sharing workouts and checking in—effectively boosts individuals' motivation and fosters a sense of belonging through two-way feedback (10). On the other hand, passive browsing provides individuals with a wealth of alternative experiences and behavioral models, which theoretically should boost their confidence. Yet, at the same time, this type of content exposes users to idealized body images and stringent fitness standards. This information overload and excessive social comparison easily trigger evaluation anxiety, which is negatively related to self-efficacy and significantly undermines their original behavioral confidence (11). Because the potential negative psychological offset of passive viewing statistically neutralizes the positive motivation derived from active interaction, the direct association between overall exercise-related social media use and exercise self-efficacy is likely canceled out. Instead, as Social Capital Theory and Psychological Capital Theory suggest, individuals must first obtain emotional support and behavioral norms through their social networks (12), which are then internalized into positive psychological resources like resilience and optimism (13). Consequently, rather than a direct association, specific indirect pathways serve as the core mechanisms influencing individuals' behavioral beliefs.

Despite the burgeoning literature on digital health behaviors, prior studies have predominantly focused on fragmented, pairwise associations—such as the isolated impact of social media on physical activity, the binary correlation between peer support and self-efficacy, or the direct link between exercise and resilience. Few studies provide a systematic, integrative framework to examine how fragmented digital interactions translate into stable internal cognitive beliefs through a continuous social-to-psychological capital chain. To address this literature gap, this research shifts the analytical focus from simple linear associations to an integrated “social-to-psychological” chain mechanism. We propose that external digital interactions do not directly and effectively influence behavioral beliefs; rather, they must be structurally embedded within interpersonal networks and subsequently internalized as individual coping resources. At the empirical level, we use PLS-SEM to map this sequential structural pathway. This study elucidates the complete structure of psychological internalization within the digital fitness ecosystem, providing a more systematic framework for understanding how fragmented digital resources ultimately translate into stable exercise self-efficacy.

## 2 Theoretical framework and hypotheses

### 2.1 Indirect-only effects of exercise-related social media use and exercise self-efficacy

According to Social Cognitive Theory, environmental factors shape individual self-efficacy through information provision and vicarious experiences. In the digital environment, exercise-related social media use serves as a salient macro-environmental resource.

On the one hand, watching workout videos, following fitness influencers, and participating in check-ins provide instructional cues and vicarious validation, which some studies suggest can inform behavioral beliefs and enhance physical activity self-efficacy (14, 15). Self-efficacy often acts as a crucial bridge to translate this digital sports knowledge into action (16). However, we theoretically propose that exercise-related social media use does not exert a significant, unmediated direct effect on exercise self-efficacy due to two competing psychological mechanisms that act in opposite directions. While serving as a cognitive enhancement tool, social media is also a highly curated space. Exposure to idealized body images inevitably triggers maladaptive “upward social comparisons” and body dissatisfaction. This induces physical anxiety, which directly counteracts the positive cognitive gains from information exposures (17). Moreover, excessive digital immersion can even deplete an individual's positive psychological capital, such as baseline resilience and efficacy (18). This competitive suppression effect implies that simply browsing online content, without being embedded in positive social connections or transformed through adaptive internal psychological traits, lacks sufficient sociopsychological influence to alter deeply ingrained perceptions of self-efficacy. Consequently, the relationship between exercise-related social media use and exercise self-efficacy is complex in nature; any substantial overall effect between the two may be achieved through indirect, chain-like transmission pathways, requiring the transformation of superficial online interactions into stable social and psychological capital. Based on these *a priori* theoretical assumptions, we propose:

**H1:** Exercise-related social media use has no significant direct association with exercise self-efficacy but exhibits a significant overall positive relationship that is fully explained by indirect pathways.

### 2.2 The indirect role of peer relationships

With the development of social media, individuals' social networks have gradually expanded from offline settings to online platforms, and social media has become a key medium for the formation of social capital (19). In sports environments, social media interactions can foster connections between people. Actions such as liking, commenting, and sharing enhance peer interactions, thereby strengthening peer relationships (20).

Peer relationships and social support play a significant role in college students' participation in physical activity. Peer support not only boosts individuals' confidence in participating in physical activities but also promotes the development of physical activity behaviors through social comparison and group norms (21). Research has directly shown that peer relationships have a significant impact on exercise self-efficacy among college students (22). It is worth noting that, in studies on adolescent depression, positive peer relationships improve mild depressive symptoms by moderating self-efficacy, enhancing adolescents' confidence in both academic and daily life (23). Therefore, exercise-related social media use may be structurally linked to college students' exercise self-efficacy by fostering peer relationships. Based on this, this study proposes the following hypothesis:

**H2:** Peer relationships serve as a significant indirect link between exercise-related social media use and exercise self-efficacy.

### 2.3 The structural pathway of psychological resilience

Psychological resilience, as an endogenous resource within an individual, refers to the ability to adapt effectively in the face of adversity; its core function is to help individuals overcome difficulties and grow through stressful and potentially traumatic events (24). As a key component of psychological capital, psychological resilience reflects an individual's ability to remain positively adaptive and persistently effortful when confronting stress, challenges, and difficulties (25). A resilient mindset helps individuals develop the fundamental elements of athletic performance, which are essential for achieving high-level athletic performance (26). Research has shown that physical exercise enhances psychological resilience by providing challenges, a sense of accomplishment, physical benefits, and social interaction, thereby boosting individuals' exercise self-efficacy (27). Psychological resilience may represent a key internal resource through which social and behavioral experiences are translated into adaptive exercise-related beliefs. Among college students, resilience has been shown to mediate the association between physical activity and emotional states, suggesting its relevance for understanding exercise-related psychological adaptation (28).

In addition to genetic factors, psychological resilience is also influenced by environmental, individual, and social factors (29). In sports environments, psychological resilience is considered a key psychological factor predicting an individual's persistence in athletic activities. Research has found that social support is a key factor related to psychological resilience. Through active interaction and sharing on social media in their academic and daily lives, college students can enhance their psychological support and emotional regulation abilities to a certain extent, thereby promoting the development of psychological resilience—a process that is largely facilitated by the mutual exchange with friends (30). As mentioned earlier, sharing one's experiences on social media is positively correlated with self-efficacy (14). From this perspective, exercise-related social media use may predict college students' self-efficacy through psychological resilience. Based on the above discussion, this study proposes the following hypothesis:

**H3:** Psychological resilience acts as an indirect pathway linking exercise-related social media use and college students' exercise self-efficacy.

### 2.4 Sequential indirect pathways involving peer relationships and psychological resilience

While Hypotheses 2 and 3 establish peer relationships and psychological resilience as distinct, parallel indirect pathways, an advanced structural hierarchy exists between these two mediators. From the perspective of integrated social and psychological capital (13, 31), these resources do not operate in theoretical isolation; rather, the external emotional support and group norms derived from social networks act as critical environmental scaffolds that stimulate the development of an individual's internal psychological capital.

Specifically, the social support derived from social relationships is continuously transformed into psychological resources for the individual, a process central to resilience development (32). When college students receive active peer reinforcement and validate their athletic identities within digital communities, the emotional support and social interaction provided enhance their sense of psychological safety and psychological adaptability, thereby directly fostering the development of psychological resilience (33). Furthermore, high-quality close friendships enable adolescents to cope effectively with stress, establishing a robust social support network that builds resilience in the face of adversity (34). Some scholars argue for a bidirectional relationship, suggesting that psychological resilience can also promote positive interpersonal interactions (35). However, in the context of digital sports participation, peer relationships serve as the primary structural antecedent. Encouragement and support from peers help individuals maintain a positive mindset when facing exercise challenges. Subsequently, psychological resilience acts as a key psychological mechanism. As Bandura conceptualized in Social Cognitive Theory, overcoming obstacles with a resilient mindset is fundamentally tied to robust self-belief (36). Thus, this enhanced resilience promotes an individual's exercise self-efficacy. Given this hierarchical transmission—where digital resources are internalized through interpersonal networks into psychological traits that reinforce self-efficacy—this study proposes the following hypothesis:

**H4:** Peer relationships and psychological resilience form a sequential structural pathway linking exercise-related social media use and exercise self-efficacy.

**Figure 1:**
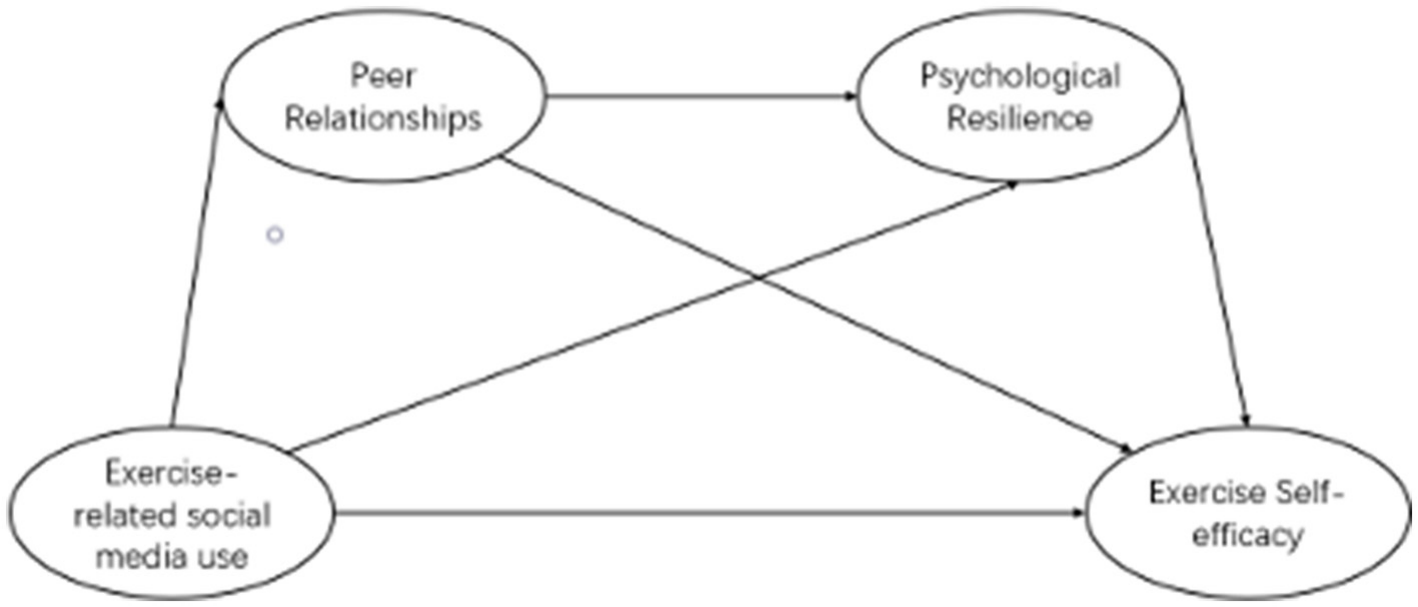
Hypothesized structural model linking exercise-related social media use, peer relationships, psychological resilience, and exercise self-efficacy.

The hypothetical model is shown in Figure 1.

## 3 Materials and methods

### 3.1 Study design

Based on a social cognitive theory framework, this study integrates social capital theory and psychological capital theory to construct a theoretical model of the structural associations: “exercise-related social media use → peer relationships → psychological resilience → exercise self-efficacy.” The study aims to identify structural associations between variables and has a predictive focus; furthermore, since the model includes a sequential structural pathway, Partial Least Squares Structural Equation Modeling (PLS-SEM) was employed for the analysis. Compared to covariance-based structural equation modeling (CB-SEM), PLS-SEM offers greater robustness and applicability when dealing with complex model structures, non-normally distributed data, and prediction-oriented studies; therefore, it is better matched to the analytical needs of this study. It is important to note that the cross-sectional design employed in this study inherently limits the ability to infer strict causal relationships between digital interactions and psychological states. Consequently, this design is primarily applicable for exploring structural associations and testing *a priori* predictive frameworks, necessitating the use of PLS-SEM to evaluate variance explanation rather than definitive causality.

### 3.2 Study population

#### 3.2.1 Source of the study population

Using stratified sampling, this study surveyed 10 comprehensive universities in the Sichuan-Chongqing region, including Sichuan University, Sichuan University of Science & Engineering, and Chongqing Jiaotong University. To ensure the representativeness of the sample, the surveyed universities covered a diverse range of academic disciplines, with participants distributed across science and engineering (43.4%), humanities and social sciences (27.6%), and sports science majors (29.0%). In addition, the distribution by academic year included freshmen (29.1%), sophomores (29.6%), juniors (22.1%), and seniors (19.2%). Male and female students accounted for 55.1% and 44.9% of the sample, respectively, with an average age of 19.51. The inclusion criteria were as follows: (1) age between 18 and 25; (2) the habit of recording or following physical activity on social media platforms; and (3) no major medical conditions that would affect physical activity. The study strictly adhered to the ethical guidelines of the Declaration of Helsinki and received formal approval from the Ethics Committee of Sichuan University of Science & Engineering prior to data collection (Approval No.: IRB202601). The approval statement is included at the beginning of the questionnaire. The informed consent form, located on the first page of the questionnaire, outlines the study's objectives, procedures, and data anonymity. Participation was entirely voluntary, and participants were informed of their right to withdraw at any time without any adverse consequences.

#### 3.2.2 Sample size

According to the latest review on PLS-SEM in software engineering research, the inverse square root method is recommended for calculating the minimum sample size (37). Compared to traditional sample size calculation methods, this approach is based on the significance of path coefficients and provides a more accurate estimate of the required sample size. Assuming a significance level of 0.05, a statistical power of 0.80, and a minimum absolute significant path coefficient (|*pmin*|) of 0.2, the sample size was calculated using the formula $n>{\left(2.486/|p_{\min}|\right)}^{2}\;$to be 155.

#### 3.2.3 Data collection

The recruitment and data collection phases were conducted concurrently from December 2025 to February 2026. After rigorously excluding invalid questionnaires (e.g., incomplete responses or responses where the same option was selected throughout), a final valid sample of 385 responses was obtained. This sample size far exceeds the minimum requirement of 155 samples calculated using the inverse square root method, ensuring sufficient statistical power and research robustness for the subsequent PLS-SEM analysis. The demographic characteristics of the respondents are detailed in Table 1.

**Table 1:** Sociodemographic characteristics of the overall sample (*N* = 385).

Variables	Frequency (%)/Mean ±standard deviation
Age	19.51 ± 1.14
Gender	
Male	212 (55.1%)
Female	173 (44.9%)
Grade	
Freshman	29.1%
Sophomore	29.6%
Junior	22.1%
Senior	19.2%
Academic discipline	
Science and Engineering	43.4%
Humanities and Social Sciences	27.6%
Sports Science Majors	30.0%
Frequency of exercise per week	2.090 ± 1.741
Daily time spent on social media	3.475 ± 1.226 (h/d)

### 3.3 Measurement of variables

#### 3.3.1 Exercise-related social media use

To accurately measure exercise-related social media use among college students, this study adapted existing mature scales to fit the specific digital physical activity context of Chinese college students. Based on previous research classifications of social media engagement, exercise-related social media use is defined as a multidimensional concept encompassing two main components: passive content consumption and active social interaction (15, 38, 39). The scale comprises three dimensions: browsing, which provides a vicarious experience of others' daily exercise routines; posting personal workout updates and photos to highlight the self-presentation of one's managed fitness image; and social feedback, which reflects interpersonal interactions and support within social networks. The scale consists of 9 items and is scored using a 5-point Likert scale, where 1 represents “strongly disagree” and 5 represents “strongly agree”; a higher score indicates a higher level of an individual's engagement with sports-related social media. Finally, the semantic equivalence of the scale items was ensured through back-translation.

During the localization adaptation process, the scale was tailored to the specific digital fitness ecosystem of Chinese college students. Culturally specific terms and behaviors, such as “sports check-ins” and “sharing on WeChat Moments or Keep,” were explicitly incorporated into the item descriptions to ensure semantic equivalence and ecological validity. Prior to the formal data collection, a pilot survey was conducted with 200 students. Exploratory Factor Analysis yielded a Kaiser-Meyer-Olkin measure of 0.865 and a significant Bartlett's test of sphericity (χ^2^ = 1,080.457, df = 36*, p* < 0.001), confirming sample adequacy. Principal component analysis identified three *a priori* dimensions: browsing, posting, and social feedback, which collectively accounted for 79.913% of the total variance, thereby demonstrating that the localized scale possesses robust structural validity. Furthermore, the revised scale had a Cronbach's alpha of 0.897, indicating strong internal consistency.

#### 3.3.2 Peer relationships, psychological resilience, and exercise self-efficacy

Peer relationships were measured using the scale developed by Yunhua (40), which originally captures dimensions of peer acceptance and peer fear. Psychological resilience was assessed using the Chinese version of the Connor-Davidson Resilience Scale (CD-RISC) (41), comprising three original dimensions: tenacity, strength, and optimism. Exercise self-efficacy was measured using the Chinese version of the Exercise Self-Efficacy Scale revised by Chen et al. (42), which assesses individuals' confidence in maintaining exercise habits across various barriers. All 3 scales were scored on a 5-point Likert scale, ranging from 1 to 5, where 1 means “strongly disagree” and 5 means “strongly agree.”

#### 3.3.3 Control variables

To ensure empirical robustness, four variables were controlled based on theoretical rationales. Gender and age were controlled due to documented demographic variances in baseline physical activity and digital media habits among emerging adults (43). According to the framework of social cognitive theory, actual behavioral performance is the primary source of self-efficacy. Therefore, weekly exercise frequency was included as a control variable (7). Daily social media usage time was controlled to explicitly isolate the specific impact of sport-related digital interactions from the general confounding effects of overall internet dependency.

Gender is coded as “male = 1, female = 0”; age, weekly exercise frequency, and daily social media usage time are treated as continuous variables.

### 3.4 Data analysis methods

PLS-SEM estimates construct parameters by indirectly addressing them through observable indicators; this approach is becoming increasingly popular for analyzing complex models (44). PLS-SEM uses measures such as the average variance extracted (AVE) to assess convergent validity, as well as the Heterotrait-Monotrait ratio of correlations (HTMT) and Fornell-Larcker discriminant validity to assess discriminant validity (45). The predictive accuracy of the model has been validated using benchmarks such as root mean square error (RMSE) and mean absolute error (MAE), which typically outperform traditional linear models (45). These characteristics make PLS-SEM an ideal choice for handling multivariate models and advancing predictive analytics.

The manufacturer of

The study therefore employs SmartPLS 4 (SmartPLS GmbH, Oststeinbek, Germany) to conduct modeling analysis and test theoretical hypotheses. By considering the overall path of the model, the comprehensive explanatory power of the predictor variables for the outcome variables is fully understood (46). Using the PLS-SEM algorithm and Bootstrapping with 5,000 resamples, we conducted significance tests on exercise-related social media use, peer relationships, psychological resilience, and exercise self-efficacy, and reported path coefficients, *t*-values, *p*-values, and confidence intervals. Additionally, we assessed the model's explanatory power and predictive ability using *R*^2^ and *Q*^2^ (47).

## 4 Results

### 4.1 Descriptive statistics and correlation analysis

The descriptive results indicate that the mean values of all variables are in the upper-middle range, suggesting that the sampled Chinese college students exhibit relatively active engagement in digital fitness environments and stable baseline levels of exercise self-efficacy (Table 2). The positive bivariate correlations among exercise-related social media use, peer relationships, and psychological resilience provide preliminary contextual evidence that active digital interaction aligns with stronger interpersonal ties and internal coping resources within the university environment. Furthermore, correlation coefficients of less than 0.70 suggest sufficient independence among the constructs. Since all variance inflation factor (VIF) values for the independent variables were below the conservative threshold of 3.3 (Table 3), the risk of multicollinearity was statistically ruled out (48).

**Table 2:** Descriptive statistics and correlation analysis (*N* = 385).

Variables	Mean	SD	1	2	3	4
Peer relationships	3.650	0.937	**1.000**			
Psychological resilience	3.680	0.880	0.521	**1.000**		
Exercise-related social media use	3.660	0.837	0.464	0.436	**1.000**	
Exercise self-efficacy	3.710	0.904	0.476	0.652	0.308	**1.000**

Bold values indicate the diagonal values representing perfect self-correlation (1.000).

**Table 3:** Results of the measurement model (factor loadings, CR, VIF, and AVE).

Latent variable	factor loadings	VIF	α	CR	AVE
Exercise-related social media use			0.900	0.918	0.555
ERSMU1	0.767	1.948			
ERSMU2	0.747	1.875			
ERSMU3	0.761	1.937			
ERSMU4	0.755	1.882			
ERSMU5	0.732	1.748			
ERSMU6	0.737	1.810			
ERSMU7	0.761	1.886			
ERSMU8	0.756	1.908			
ERSMU9	0.688	1.617			
Peer Relationships			0.865	0.899	0.597
PR1	0.768	1.750			
PR2	0.783	1.925			
PR3	0.784	1.836			
PR4	0.761	1.730			
PR5	0.762	1.724			
PR6	0.777	1.803			
Psychological resilience			0.913	0.928	0.562
RES1	0.721	1.811			
RES2	0.701	1.689			
RES3	0.781	2.146			
RES4	0.732	1.795			
RES5	0.789	2.137			
RES6	0.752	1.925			
RES7	0.742	1.874			
RES8	0.769	2.022			
RES9	0.787	2.146			
RES10	0.719	1.760			
Exercise Self-Efficacy			0.927	0.937	0.533
ESE1	0.761	2.068			
ESE2	0.706	1.814			
ESE3	0.714	1.833			
ESE4	0.746	2.011			
ESE5	0.703	1.796			
ESE6	0.761	2.053			
ESE7	0.728	1.960			
ESE8	0.722	1.855			
ESE9	0.722	1.852			
ESE10	0.734	1.950			
ESE11	0.740	1.937			
ESE12	0.749	2.032			
ESE13	0.705	1.768			

ERSMU, Exercise-related social media use; PR, Peer relationships; RES, Psychological resilience; ESE, Exercise self-efficacy.

### 4.2 Model fit tests

**Figure 2:**
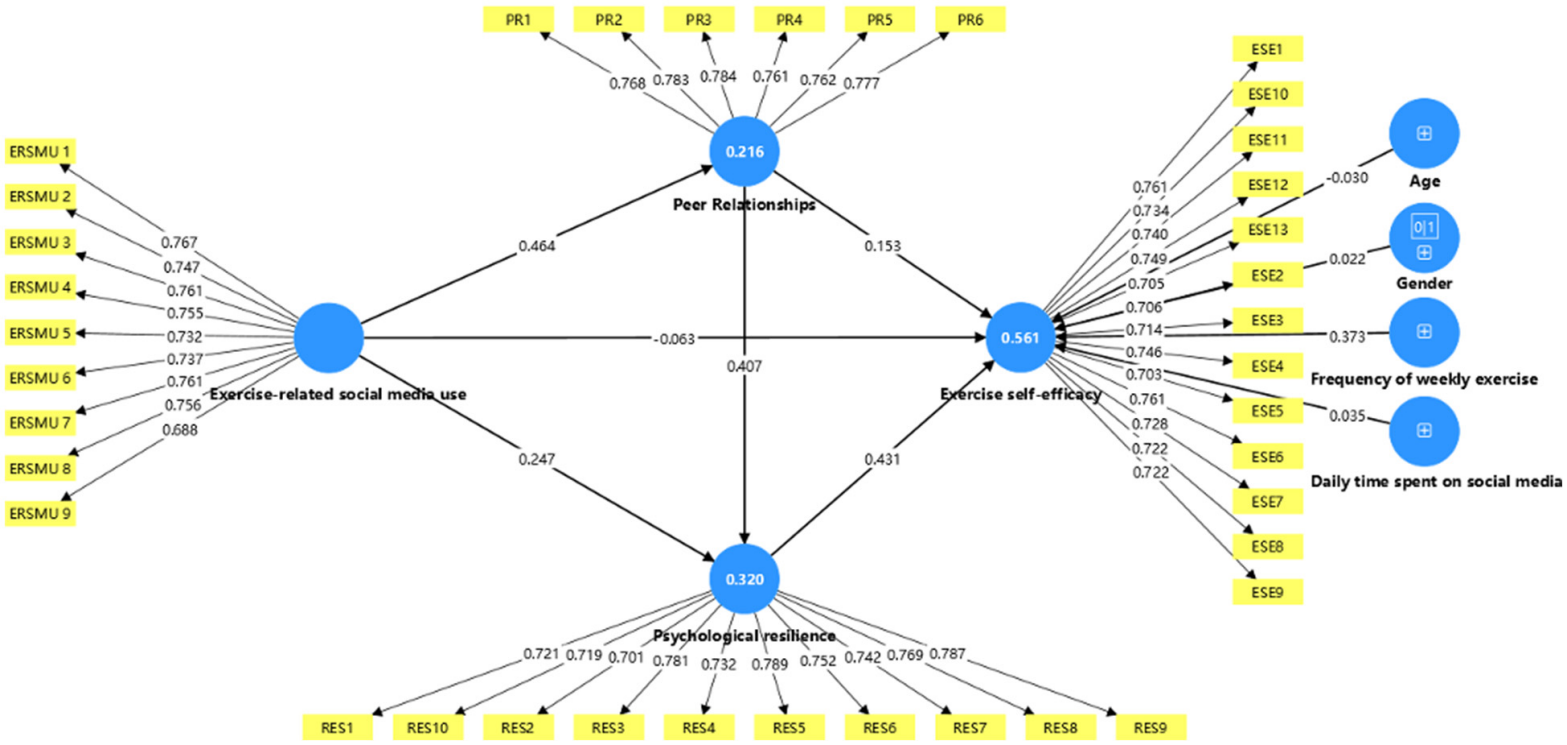
PLS-SEM algorithm results (measurement and structural model). Values on the outer arrows indicate factor loadings, values on the inner arrows indicate path coefficients, and values inside the blue circles represent the explained variance (*R*^2^). ERSMU, Exercise-related social media use; PR, Peer Relationships; RES, Psychological Resilience; ESE, Exercise Self-efficacy.

Factor loadings, Cronbach's alpha, and composite reliability (CR) coefficients were evaluated to assess the reliability and internal consistency of the latent constructs within the university physical activity context. With all factor loadings exceeding the 0.50 threshold, the individual behavioral and psychological items demonstrate acceptable reliability in operationalizing their respective sub-dimensions among college students (Table 3) (49). Cronbach's alpha and CR coefficients for all variables exceeded 0.70, confirming high internal consistency across all localized measurements. Furthermore, average variance extracted (AVE) values greater than 0.50 demonstrate acceptable convergent validity, indicating that the latent constructs successfully capture the majority of the variance in their observable indicators (50). The PLS-SEM algorithm results, illustrating the measurement model's factor loadings and the explained variance (*R*^2^) of the endogenous constructs are presented in Figure 2.

The model's discriminant validity was confirmed via the Heterotrait-Monotrait (HTMT) ratio (Table 4), with all ratios below 0.85, establishing that exercise-related social media use, peer relationships, psychological resilience, and exercise self-efficacy represent distinct empirical concepts within this demographic (49). Finally, the Standardized Root Mean Square Residual (SRMR) value of 0.039 fell safely below the 0.08 threshold, confirming a close objective fit between the hypothesized theoretical framework and the empirical survey data (51).

**Table 4:** Discriminant validity of the model (HTMT).

Variables	PR	RES	ERSMU	ESE
PR				
RES	0.585			
ERSMU	0.524	0.479		
ESE	0.528	0.706	0.336	

ERSMU, Exercise-related social media use; PR, Peer relationships; RES, Psychological resilience; ESE, Exercise self-efficacy.

### 4.3 Path analysis

Before formally beginning the path coefficient analysis, we performed a diagnostic check for multicollinearity using the variance inflation factor (VIF). The results show that the VIF values for each path in the endogenous structure of the model range from 1.000 to 1.628, which is below the critical value of 3.3, indicating that there is no multicollinearity (52).

**Figure 3:**
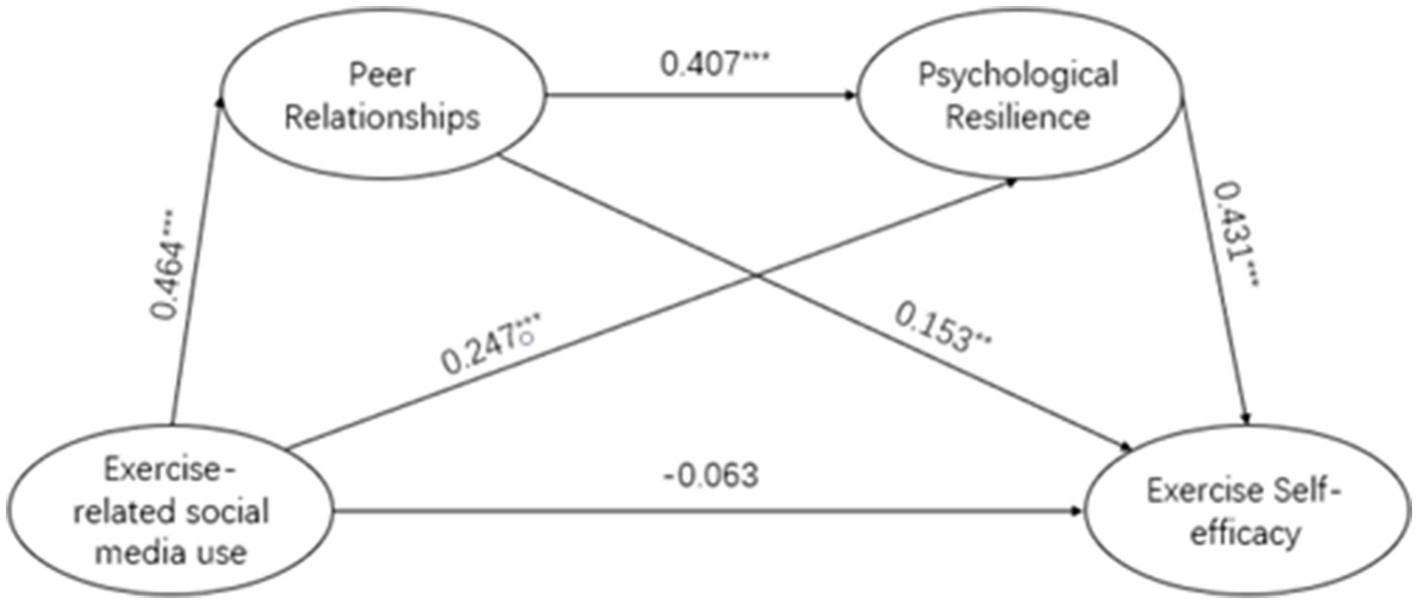
Structural model results with path coefficients and significance. *p < 0.05, **p < 0.01, ***p < 0.001. Non-significant paths are presented without markers.

Accordingly, this study uses Bootstrapping in SmartPLS 4 to test the hypothesized paths; the results are shown in Table 5 and the structural path coefficients are illustrated in Figure 3. Exercise-related social media use had a significant positive association with peer relationships (β = 0.464, *p* < 0.05) and psychological resilience (β = 0.247, *p* < 0.05), indicating that digital interactions are robustly tied to both external social capital and internal psychological assets among college students. Crucially, within the relational sequence, peer relationships positively predicted psychological resilience (β = 0.407, *p* < 0.05) and exercise self-efficacy (β = 0.153, *p* < 0.05), while psychological resilience acted as a strong proximate predictor of exercise self-efficacy (β = 0.431, *p* < 0.05). The direct association between exercise-related social media use and exercise self-efficacy was not significant [95% CI: (−0.151, 0.021)]; however, in the overall structural model, exercise-related social media use has a significant positive relationship with exercise self-efficacy (β = 0.196, *p* < 0.05). This indicates that the association of exercise-related social media use with exercise self-efficacy does not occur directly but is fully accounted for by the indirect pathways.

**Table 5:** Path coefficients and hypothesis tests.

Hypothesis	Path	β	*t*-value	*p*-value	95% Confidence interval	VIF
	ERSMU → ESE	−0.063	1.442	0.149	(−0.151, 0.021)	1.500
	ERSMU → PR	0.464	11.637	0.000	(0.388, 0.542)	1.000
	ERSMU → RES	0.247	5.284	0.000	(0.152, 0.338)	1.275
	PR → RES	0.407	8.621	0.000	(0.314, 0.499)	1.275
	PR → ESE	0.153	3.439	0.001	(0.065, 0.239)	1.537
	RES → ESE	0.431	10.056	0.000	(0.346, 0.515)	1.628
Overall path	ERSMU → ESE	0.196	4.562	0.000	(0.111, 0.281)	

ERSMU, Exercise-related social media use; PR, Peer relationships; RES, Psychological resilience; ESE, Exercise self-efficacy.

In addition to the direct association, this study further tested the indirect roles of peer relationships and psychological resilience in the relationship between exercise-related social media use and exercise self-efficacy. As shown in Table 6, the 95% confidence intervals for all three indirect paths do not include zero. The path coefficients for the “exercise-related social media use → peer relationships → exercise self-efficacy” and “exercise-related social media use → psychological resilience → exercise self-efficacy” pathways were both significant (β = 0.071, *p* < 0.05; β = 0.106, *p* < 0.05). The indirect paths via peer relationships and psychological resilience were significant, and both H2 and H3 were confirmed. The sequential indirect path of “exercise-related social media use → peer relationships → psychological resilience → exercise self-efficacy” (β = 0.081, *p* < 0.05) was also significant, confirming Hypothesis H4. In the context of student health behaviors, these results provide preliminary confirmation that digital media exposure lacks a direct, unmediated impact on efficacy beliefs; instead, its positive association is fully realized through a sequential transmission from social networks to individual psychological resources.

**Table 6:** Indirect associations.

Path	Indirect path	*t*-value	*p*-value	95% Confidence Interval
ERSMU → PR → ESE	0.071	3.189	0.001	(0.029, 0.117)
ERSMU → RES → ESE	0.106	4.700	0.000	(0.064, 0.154)
ERSMU → PR → RES → ESE	0.081	5.330	0.000	(0.055, 0.115)
Total Indirect Path	0.259	8.374	0.000	(0.199, 0.322)

ERSMU, Exercise-related social media use; PR, Peer relationships; RES, Psychological resilience; ESE, Exercise self-efficacy.

### 4.4 Control variable test

The structural analysis revealed that weekly exercise frequency had a highly significant positive association with exercise self-efficacy [β = 0.373, *p* < 0.05, 95% CI: (0.304, 0.442)]. However, the associations of gender, age, and daily social media usage time were not statistically significant, as shown in Table 7. In the context of student health behaviors, this finding underscores that regardless of the virtual support acquired through social media, actual physical mastery experiences in the real world remain the fundamental anchor for sustaining individual efficacy beliefs. Conversely, the non-significant associations for daily social media time confirm that efficacy accumulation depends on targeted sport-specific media interaction rather than baseline screen exposure.

**Table 7:** Control variables.

Path	β	*t*-value	*p*-value	95% Confidence Interval
Gender	0.022	0.320	0.749	(−0.114, 0.161)
Age	−0.030	0.863	0.388	(−0.098, 0.037)
Frequency of weekly exercise	0.373	10.681	0.000	(0.304, 0.442)
Daily time spent on social media	0.035	0.961	0.336	(−0.038, 0.108)

### 4.5 Evaluation of the model's explanatory power and predictive capability

After conducting significance tests on the path coefficients, this study further evaluated the theoretical explanatory power and out-of-sample predictive ability of the structural model. The model's explained variance (*R*^2^) for the intermediate variables—peer relationships and psychological resilience—was 0.216 and 0.320, while its explained variance (*R*^2^) for the core dependent variable, exercise self-efficacy, reached 0.561, indicating a level of theoretical explanation that is slightly above average (49).

An analysis of out-of-sample predictive ability using the PLSpredict (*k* = 10, *r* = 10) revealed that the overall $Q_{predict}^{2}$ for the latent factor levels of exercise self-efficacy (0.336), peer relationships (0.208), and psychological resilience (0.184) were all greater than 0, preliminarily establishing that the structural model exhibits good predictive validity at the macro level. A comparison of the root mean square error (RMSE) between the PLS-SEM model and the simple linear regression (LM) model revealed that the PLS-SEM prediction errors for the core variable—measures of exercise self-efficacy—were consistently lower than those of the LM model (Table 8). Similarly, for the intermediate variable—peer relationships, the PLS-SEM prediction errors for most of its measures were lower than those of the LM model. Both variables demonstrated robust, moderate-to-high levels of predictive accuracy. Regarding psychological resilience as an intermediate variable, only a minority of its indicators yielded lower prediction errors in PLS-SEM compared to the LM model, indicating weak out-of-sample predictive power for this specific construct. From a preliminary perspective, as a deep-seated and broad psychological trait, psychological resilience is influenced by complex life events that extend beyond the specific context of sports-related digital interactions. Overall, the core variable of the model's ultimate research objective, exercise self-efficacy, achieved a high level of predictive power, indicating that the model explains the formation of individual exercise beliefs and retains substantial predictive value even in out-of-sample data (49, 53).

**Table 8:** Estimates of predictive ability.

Key Metrics	${\text{Q}}_{\text{predict}}^{2}$	PLS-SEM (RMSE)	LM (RMSE)
Exercise self-efficacy			
ESE1	0.201	**0.898**	0.911
ESE2	0.171	**0.887**	0.893
ESE3	0.185	**0.868**	0.875
ESE4	0.187	**0.888**	0.900
ESE5	0.162	**0.917**	0.918
ESE6	0.199	**0.894**	0.894
ESE7	0.169	**0.902**	0.916
ESE8	0.168	**0.874**	0.885
ESE9	0.161	**0.873**	0.882
ESE10	0.166	**0.906**	0.912
ESE11	0.179	**0.877**	0.887
ESE12	0.189	**0.903**	0.904
ESE13	0.182	**0.852**	0.867

Bold values represent the lower Root Mean Square Error (RMSE) values between the PLS-SEM and LM models, which indicate better predictive accuracy.

## 5 Discussion

### 5.1 Why the direct association between exercise-related social media use and ESE is not significant

This study found that exercise-related social media use had no significant direct association with exercise self-efficacy, but its overall path was significant and was fully explained by indirect pathways through peer relationships and psychological resilience. This finding diverges from early linear digital health models, which often posited a straightforward positive impact of fitness app usage on sports confidence (54, 55). These findings suggest that isolated exposure to digital contextual resources possesses limited explanatory power for individuals' beliefs about their abilities; rather, its relevance emerges primarily within a structural network involving social and psychological factors.

As our study explains, fitness content on social media often displays idealized body images and high-intensity workouts; while this can motivate people to exercise, it can also easily trigger body image anxiety or feelings of inadequacy, thereby directly counteracting its positive impact on a sense of efficacy (56, 57). Consistent with a recent meta-analysis (57), this superficial phenomenon of digital exposure does not necessarily manifest as stable psychological capital. Without active social embeddedness, it may even trigger comparison-induced anxiety, which counteracts positive associations (58). From a mechanistic perspective, social media does not provide individuals with a sense of self-efficacy itself, but rather a form of “digital contextual resource.” These resources primarily take the form of vicarious experiences and behavioral demonstrations. However, passive information exposure or “shallow digital engagement” (e.g., merely scrolling or consuming fitness content without active interaction) lacks the requisite psychosocial density to directly transform deep-seated self-efficacy beliefs (59). The fact that the path coefficient for social media usage time among the control variables was not significant further supports this view: while simply browsing fitness content, following fitness influencers, and watching workout videos can spark interest in exercise in the short term, this interest will not endure unless it progresses to the stages of social interaction, real-world support, and psychological internalization. Therefore, our study confirms that social media does not provide individuals with a sense of self-efficacy itself. Rather, it functions as a “digital contextual resource” taking the form of vicarious experiences and behavioral demonstrations.

The absence of a significant direct pathway from exercise-related social media use to exercise self-efficacy is theoretically meaningful. It suggests that digital content alone may be insufficient to overcome effort-related barriers to physical activity (9). Because exercise-related social media use is commonly embedded in screen-based sedentary contexts, future research and theoretical models must distinguish passive exposure from cognitively active, socially interactive, and content-relevant digital engagement (60, 61). When social media use remains passive rather than socially interactive or psychologically internalized, it lacks the motivational activation needed to drive behavioral beliefs.

### 5.2 Peer relationships as a crucial explanatory node in digital sports participation

The study findings indicate that peer relationships play a significant indirect role between exercise-related social media use and exercise self-efficacy. While previous literature often positions social media primarily as an information acquisition tool for health knowledge (62), our findings demonstrate that its core value in shaping behavioral beliefs lies in structural social embeddedness. The value of exercise-related social media use isn't in directly boosting individuals' confidence in their physical abilities through mere exposure, but rather in strengthening their sense of community and social support through interactive features like likes, comments, and mutual check-ins. For college students, exercise is often not a purely individual activity, but rather a social practice deeply influenced by peer dynamics, group norms, and emotional contagion (63). Social media has broken down physical barriers and established supportive sports communities through online interaction, with the core value in the accumulation of social support (39).

When college students consistently participate in sports-related activities, the resulting sense of social connection makes it easier for them to maintain consistency in their exercise routines (e.g., perceiving that “someone is sticking with it alongside me”). In addition, this socially embedded logic perfectly aligns with the significant positive association of the control variable “weekly exercise frequency”: an individual's firsthand mastery experience remains the most potent source of self-efficacy. The alternative experiences and social reinforcement provided by digital platforms must be complemented by real-life peer support and regular physical exercise to truly reinforce a sense of self-efficacy.

### 5.3 Psychological resilience as the proximate explanatory asset for self-efficacy

This study also found that psychological resilience serves as a significant intermediate link, with its specific path coefficient higher than that of the isolated peer relationships path. Existing sports psychology literature typically treats peer relationships and psychological resilience as parallel or independent predictors of exercise behavior (23, 24). By contrast, our model confirms that resilience acts as a proximate, internalized asset that must be activated by antecedent variables.

Exercise itself inherently involves a host of challenges, including fatigue, inertia, scheduling conflicts, and physical discomfort (64). Psychological resilience represents an individual's ability to maintain focus and restore action in challenging situations (24). In the digital fitness context, positive feedback, phased achievement displays, and group atmosphere on social media act as critical psychological buffers for individuals encountering sports setbacks (65). Importantly, this finding expands upon the prevailing “digital depletion model,” which argues that internet addiction or excessive screen time is negatively correlated with psychological resilience (35, 66). Our research indicates that in specific, health-oriented contexts, active and community-based digital engagement serves as a catalyst rather than a depleter of psychological resilience, structurally bridging the gap between external digital support and internal exercise self-efficacy. It is worth noting that while PLSpredict results indicated weak predictive power for psychological resilience, this is theoretically plausible. As a deep-seated, endogenous form of psychological capital, resilience's variance cannot be fully captured by external social media interactions alone (67).

### 5.4 The Sequential associations from social capital to psychological capital to belief in one's abilities

The PLS-SEM analysis illustrates that digital interactions, peer relationships, and psychological resilience operate not as independent factors, but through a chain-based mediation pathway (ERSMU → PR → RES → ESE). This configuration corroborates Luthans and Youssef-Morgan's psychological capital framework (13), indicating that the external social capital accumulated from digital networks must be internalized into psychological resilience to effectively solidify exercise self-efficacy.

The integrated structural model demonstrates substantial explanatory power (*R*^2^ = 0.561) for exercise self-efficacy. Traditional physical activity models, which often rely solely on physical environmental variables or isolated psychological traits, typically yield a low to moderate explanatory variance, often ranging from 20 to 40%, with classical Social Cognitive Theory models averaging around 31% (68). The robust variance explained in our study underscores that digital contextual resources must be structurally nested within both social and psychological capital to maximize behavioral belief explanations. Furthermore, addressing contemporary methodological calls in behavioral modeling (53), the robust out-of-sample predictive validity (Q^2^predict > 0 and lower RMSE values compared to LM models) confirms that the sequential pathway from social to psychological capital possesses substantial predictive capability for novel data. This proves that the identified structural synergy is a widely applicable mechanism rather than an artifact of in-sample statistical overfitting (49).

## 6 Implications and recommendations

### 6.1 Theoretical contributions

This study offers distinct theoretical contributions. First, correcting the linear perception of social media influence: By confirming that exercise-related social media use operates through a fully indirect pathway, this study dispels the simplified empirical assumption that information exposure directly translates into behavioral beliefs. It extends Social Cognitive Theory by empirically delineating the “competitive suppression effect” within digital environments. Second, expanding the contextual interpretation of structural theories: By structurally integrating social capital and psychological capital, we mapped a sequential “outside-in” internalization mechanism. This bridges the theoretical gap between fragmented digital sociology studies and endogenous sports psychology, proving that exercise-related social media use, peer relationships, and psychological resilience function concurrently as an integrated system rather than isolated predictors.

### 6.2 Practical insights

Based on the verified structural model, this study provides actionable insights. First, university-based exercise promotion on social media should therefore prioritize interactive, enjoyable, content-relevant, and socially rewarding experiences, because positive affective experiences may act as more proximal drivers of physical activity engagement than health information alone (69). Second, optimizing algorithms to reduce comparison: fitness platform developers should optimize their algorithms to prioritize realistic, peer-supported workout content over extreme influencer imagery to mitigate upward comparison-induced anxiety. Finally, emphasizing the cultivation of psychological resources: campus psychological counseling centers should incorporate sports-targeted resilience-building modules, equipping students with emotional regulation strategies to independently internalize social support into enduring exercise persistence during periods of fatigue.

## 7 Limitations and future research

This study examined the relationships among exercise-related social media use, peer relationships, psychological resilience, and exercise self-efficacy, and confirmed a fully indirect structural pathway; however, certain limitations remain.

Regarding measurement methodology: all data were collected via self-report questionnaires, making them inevitably susceptible to social desirability bias. Specifically, in a university setting, individuals who demonstrate “self-discipline and health-consciousness” are held in high regard. Participants may unconsciously exaggerate their self-reported exercise self-efficacy to project confidence and overestimate their active social media engagement to conform to positive peer norms. Future research needs to shift from subjective assessments to multimodal objective data. For example, researchers should utilize digital phenotyping via platform APIs to accurately capture actual online interaction frequencies, while using wearable accelerometers or smartwatches to continuously track actual physical activity levels, thereby cross-validating self-reported psychological indicators.

Regarding theoretical boundary conditions: this study evaluated exercise-related social media use as a monolithic construct without exploring the potential moderating effects of specific exercise modalities and social media platform types. For instance, team-based sports inherently foster stronger real-world peer relationships than solo aerobic exercises, which may alter the reliance on digital social capital. Similarly, acquaintance-based platforms may yield higher-quality, trust-based social support, whereas stranger-based public fitness communities might trigger more intense upward social comparison and body anxiety. Future research should treat these typologies as moderating variables. Conducting multi-group SEM to compare different platform users and exercise types will help establish the precise boundary conditions under which this chain-mediation mechanism operates most effectively.

In terms of research design: the cross-sectional nature of the data limits the ability to establish a strict chronological sequence and absolute causal relationships among variables. The transformation from social capital to psychological capital is inherently a dynamic, time-dependent process. Future research should adopt rigorous longitudinal designs, specifically employing Cross-Lagged Panel Models. By tracking cohorts of college students across multiple distinct time points, researchers can dynamically monitor how fluctuations in digital engagement temporally precede shifts in peer support networks and subsequent resilience, thus providing robust causal evidence for the sequential mechanism.

Finally, while the sample size met rigorous statistical thresholds, it was geographically restricted to universities in the Sichuan-Chongqing region. Factors such as regional digital infrastructure, cultural background, and socioeconomic disparities might influence digital health behaviors. Future studies should employ stratified national sampling across diverse university typologies and countries to verify the cross-cultural generalizability of this PLS-SEM structural framework.

## 8 Conclusions

The study found that exercise-related social media use has no significant direct association with exercise self-efficacy, but its overall association is significant and is fully explained by indirect paths, with “peer relationships → psychological resilience” serving as the key explanatory pathway. This demonstrates that exercise-related social media use is positively associated with social capital, which structurally aligns with psychological capital, ultimately correlating with the formation of individuals' beliefs about their own capabilities. These findings deepen our theoretical understanding of how social and psychological factors interact to shape physical activity in digital contexts, offering new insights for physical education practices in higher education institutions.

## Data Availability

The raw data supporting the conclusions of this article will be made available by the authors, without undue reservation.
